# Knowledge about schizophrenia test: the Chinese Mandarin version and its sociodemographic and clinical factors

**DOI:** 10.1186/s12888-023-04822-9

**Published:** 2023-07-24

**Authors:** Ming Wang, Miaomiao Zhao, Wufang Zhang, Wenxiu Li, Rui He, Ruoxi Ding, Ping He

**Affiliations:** 1 School of Public Health, Peking University, 38 Xue Yuan Road, Haidian District, Beijing, 100191 China; 2 China Center for Health Development Studies, Peking University, 38 Xue Yuan Road, Haidian District, Beijing, 100191 China; 3 Shanghai Mental Health Center, Shanghai Jiao Tong University School of Medicine, Shanghai, 200030 China; 4 Center for Mental Health Management, China Hospital Development Institute, Shanghai Jiao Tong University, Shanghai, 200030 China; 5 NHC Key Laboratory of Mental Health (Peking University), Peking University Sixth Hospital, Peking University Institute of Mental Health, National Clinical Research Center for Mental Disorders (Peking University Sixth Hospital), Beijing, China; 6 Beijing Haidian Psychological Rehabilitation Hospital, Beijing, China

**Keywords:** Schizophrenia, Caregivers, Knowledge about Schizophrenia Test, Chinese-version

## Abstract

**Background:**

Schizophrenia is a chronic, complex, and severe mental disorder and caregivers having knowledge about it can help improve patient adherence to treatment. This study aims to translate the Knowledge About Schizophrenia Test (KAST) into a Chinese Mandarin version and test it among caregivers to validate its reliability and reproducibility, as well as to determine its associated sociodemographic factors and clinical factors.

**Methods:**

The project surveyed 160 patients with schizophrenia and their caregivers at four community health facilities in Beijing, China, from January 2022 to February 2022. All patients and caregivers completed the sociodemographic questionnaire, and caregivers also completed the Chinese-version KAST, and 143 of these caregivers completed the Chinese-version KAST again 2–4 weeks later.

**Results:**

The mean (SD) of the caregiver score was 11.49 (± 3.13). After item analysis, there was acceptable internal consistency among the 17 items in the Chinese version (KR-20 coefficient 0.702). The intraclass correlation coefficient in the retest (0.686) was statistically significant. Gender, educational attainment, marital status, relationship with the patient, and occupational status were associated with the KAST score.

**Conclusion:**

The findings demonstrate that the Chinese-version KAST is a reliable and reproducible instrument that can measure knowledge about schizophrenia and is valid to be applied in schizophrenia research.

## Background

Schizophrenia, a complex and severe mental disorder, is characterized by positive symptoms, negative symptoms, and a series of cognitive and social impairments [1, 2]. The prodromal phase (i.e., before the first psychotic episode) of schizophrenia is usually thought to begin in early adolescence and manifest as a mild decline in cognitive and social functioning [3]. The adverse health outcomes impose a heavy burden on patients with schizophrenia, their families, and society. In China, the patient’s family members often assume the caregiver’s role. Previous studies have shown that knowledge of the psychiatric disorder, treatment outcomes, and social consequences are associated with insight and that lack of insight leads to medication nonadherence [4], which in turn is associated with poor prognosis [5, 6]. Therefore, assessing the knowledge of patients and their caregivers about schizophrenia is crucial for the rehabilitation intervention of patients.

For the assessment of schizophrenia knowledge, several scales have been developed to measure caregivers’ or patients’ knowledge of schizophrenia, including the Knowledge About Schizophrenia Interview (KASI)[7], Knowledge About Schizophrenia Questionnaire(KASQ)[8], Knowledge of Schizophrenia (KOS)[9], and Knowledge About Schizophrenia Test (KAST)[10], which are typically used to measure mental health literacy, including knowledge of disease etiology, diagnosis, prevalence, symptoms, comorbidities, treatment, side effects, and health services. One of the original KAST was 21 multiple-choice questions on knowledge of schizophrenia, later revised to 18-KAST [10], and a test in Portugal selected 17 multiple-choice questions [11], studies have shown that using the KAST to measure knowledge of schizophrenia is valid and reliable. At present, there is no Chinese-version (Mandarin) KAST in China, so it is very important to examine the reliability and validity of the Chinese version of KAST.

This study aimed to translate the Knowledge About Schizophrenia Test (KAST) into a Chinese Mandarin version and apply it to caregivers of patients with schizophrenia in China to evaluate its reliability, as well as predict which factors were associated with caregivers’ scores on the Knowledge About Schizophrenia Test.

## Methodology

### Subjects and procedure

A cross-sectional survey was conducted at four community health facilities in Beijing, China, from January 2022 to February 2022. Inclusion criteria for patients with schizophrenia are (1) age 18 or above; (2) initial diagnosis of schizophrenia by a psychiatrist according to ICD-10 [12] criteria; and (3) acute or stable phase. And the exclusion criteria are patients with severe physical illness, mental retardation, co-morbid bipolar disorder, and major depressive disorder.

Inclusion criteria for sampling caregivers are (1) age 18 or above; and (2) able to understand, read, and communicate with the investigators in Chinese. And the exclusion criteria are (1) caregivers who did not speak Chinese or could not communicate effectively; (2) caregivers with any mental illness; and (3) caregivers who provided care for more than one family member with a chronic physical or mental illness.

First, all subjects were informed of the purpose of the study and informed that written informed consent was obtained and signed before participation. Second, the researcher presented the overall content of the questionnaire to each eligible subject, and then the subjects completed the questionnaire by self-filling. Finally, quality control investigators check the answers for accuracy, completeness, and consistency.

### Choice of the assessment instrument

The KAST was chosen as the instrument for this study. It is a short multiple-choice knowledge test about schizophrenia, with each multiple-choice question containing a word stem and five response options (A through E, one correct answer, and four interfering items). Initially, 12 items were developed, including six domains (causes, symptoms, diagnosis, course, treatments, and self-help), each comprising two items. This was increased to 21 items after expert review. Based on item analysis and KR-20 reliability statistics, the final English version contains 18 items. The 18-item version demonstrated good internal consistency reliability and validity when tested with a sample population of lay community members, caregivers of people with schizophrenia, and mental health professionals [10].

### Translation and cross-cultural adaptation

The translation and cross-cultural adaptation of the original KAST questionnaire followed the guidelines described by Beaton et al [13]. The procedure was carried out in five steps. The first step was to conduct a forward translation. The original English version of KAST was independently translated into Chinese by two Chinese translators familiar with English culture but native speakers of Chinese (Mandarin), one familiar with mental illness expertise and the other unfamiliar with mental illness. In the second step, the translations were synthesized. The two translators and a psychiatrist with a bilingual background synthesized the two versions to initially generate a universally translated Chinese version. Step 3: Reverse translation. Starting with the preliminary Chinese version, native English-speaking translators who had never been exposed to the KAST questionnaire translated it back into the original version. Step 4: Expert committee review. Two psychiatrists and a psychologist were invited to integrate all translated versions and develop the final Chinese-version KAST for field testing. step 5: pretesting the Chinese-version KAST. PriBeforee the official start of testing,10 caregivers of people with schizophrenia were invited to test the final Chinese-version KAST to again assess its fluency and difficulty of comprehension. The expert committee made appropriate adjustments based on the pretest results to form the final Chinese-version KAST and agreed to use it with a larger group of patients to assess its validity and reliability.

### Procedures for consistency and reproducibility

Since the KAST uses a dichotomous scoring method and the Kuder-Richardson 20 (KR-20) [14] applies to the measurement of dichotomous variables, the internal consistency evaluation of the Chinese-version KAST in this study used the KR-20 reliability coefficient. Like Cronbach’s alpha [15], with values between 0 and 1, a value of 0.70–0.90 for internal consistency is generally acceptable [16]. To assess reproducibility, the application was repeated (test-retest) for 143 of these caregivers 2 to 4 weeks after the first application.

### Validity analysis

Factor analysis and principal component maximum variance rotated factor analysis was used to examine the structural validity of the Chinese Mandarin version of KAST. We used the Kaiser-Meyer-Olkin (KMO) test, and Bartlett’s sphere test to determine whether items were suitable for factor analysis.

### Other variables and measures

Various sociodemographic variables were assessed, including age, gender, marital status, educational attainment, occupational status, household financial issue, and relationship with the patient. Patients’ clinical factors assessed only the duration of schizophrenia. These variables were reported by patients or their caregivers. Household financial issues were measured by a question: is your family experiencing financial difficulties? Response options include not at all, lighter, fair, and more severe. Duration of illness(years) was calculated by the self-reported onset time and investigation time of the patients and was divided into 4 groups: 14 or below,15–24,25–34, and 35 or above.

### Data analysis methods

Descriptive analyses were performed to present sample characteristics. The categorical variables were presented as counts (percentages), while the continuous variables were presented as means (SD). Intraclass correlation coefficient (ICC) and Pearson correlation coefficient were used to assess the retest reliability. The Spearman correlation coefficient was used for correlation analysis. Multivariate linear regression analysis was used to assess the sociodemographic and clinical factors that were exactly associated with the KAST score. All tests were two-tailed with a statistical significance level of p < 0.05. All statistical analyses were performed using STATA software version 17.0.

## Result

### Sociodemographic and clinical factors

The sociodemographic characteristics of the sample are shown in Table 1. Of the 160 caregivers surveyed, 81 (50.63%) were male and ranged in age from 18 to 94 years (Mean = 60.63, SD = 14.07). The majority had a high school education or above (n = 91, 56.88%), and the vast majority (80.63%) were married. More than half of the caregivers were retired (65.63%), and only a small proportion (9.38%) of the families faced serious financial difficulties, with the majority of caregivers being parents (36.25%) or spouses (33.75%).

Of the 160 patients with schizophrenia surveyed, 82 (51.25%) were female and ranged in age from 24 to 91 years (M = 53.12, SD = 13.24). The majority had a high school education or above (n = 105, 65.63%), and nearly half (47.50%) were married. Nearly one-third of the patients were unemployed (35.00%), and the duration of illness ranged from 0 to 56 years (M = 25.89, SD = 11.95).

**Table 1:** Sociodemographic and clinical factors

	Caregivers(CG) (n = 160)n(%)	Patients (n = 160)n(%)
**Gender** Male Female	81(50.63) 79(49.38)	78(48.75) 82(51.25)
**Age(years)** 44 or below 45–54 55–64 65 or above	Mean(SD) = 60.63(14.07) 18(11.25) 32(20.00) 48(30.00) 62(38.75)	Mean(SD) = 53.12(13.24) 44(27.50) 41(25.63) 44(27.50) 31(19.38)
**Educational attainment** Middle school or below High school or Junior college College or above	69(43.13) 32(20.00) 59(36.88)	55(34.38) 61(38.13) 44(27.50)
**Marital status** Unmarried Married Other(Divorced/Widowed)	11(6.88) 129(80.63) 20(12.50)	64(40.00) 76(47.50) 20(12.50)
**Occupational status** Retired Unemployed Employed	105(65.63) 13(8.13) 42(26.25)	70(43.75) 56(35.00) 34(21.25)
**Household financial issue** Not at all Lighter Fair More serious	33(20.63) 34(21.25) 78(48.75) 15(9.38)	
**Relationship with the patient** Spouse Parent Children Others(e.g., Siblings)	54(33.75) 58(36.25) 18(11.25) 30(18.75)	
Duration of illness(years) 14 or below 15–24 25–34 35 or above		Mean(SD) = 25.89(11.95) 26(16.25) 56(35.00) 38(23.75) 40(25.00)

### Translation and cross-cultural adaptation

The translators did not have significant disagreements in the translation of KAST, and the accuracy of the initial translation was demonstrated through reverse translation. After reviewing the combined translations, the expert committee suggested that question 17 is not applicable, because of different social backgrounds, patients and their families can obtain relevant information and support for schizophrenia from some formal and more official organizations such as the Chinese Medical Association and the CDC. In question 9, “FBI” was changed to “Police”. After pretesting 10 caregivers of people with schizophrenia, the results reported no difficulty in understanding the phrases and sentences in KAST, so the 17-item Chinese version of KAST did not need to be adjusted. The final Chinese version of KAST had a maximum total score of 17, including the following relevant items: etiology, symptoms, diagnosis, treatment, evolution and outcome, and management.

### Consistency and reproducibility

#### Internal consistency

The KR-20 reliability coefficient was used to evaluate the correlation between the entries of each dimension of the KAST and the overall correlation between the entrances of the whole scale. The results showed that the KR-20 coefficient was 0.702, then the Chinese-version KAST’s internal consistency was considered acceptable. There are no redundant items. An average inter-item correlation of 0.15 to 0.50 is recommended [17]. The internal correlations for all items of the 17-item KAST were above 0.15 (range = 0.17–0.48), and the mean of the 17 correlations was 0.29, all meeting expectations.

#### Question analysis of the KAST

The study found that the mean score for the KAST questions was 11.49. 90.63% of caregivers answered question 3 correctly. 86.25% of questions 11 and 12 were both correct. 83.75% of caregivers answered question 5 correctly. Question 10 had a correct response rate of 82.5%. Only 41.88%, 45%, 46.25%, and 49.38% of the caregivers answered questions 16, 2, 8, and 9 correctly, respectively(Table 2). Specifically, more than half of the caregivers were unaware of the new “atypical” medicines used to treat schizophrenia, the common symptoms of schizophrenia, and the causes of schizophrenia.

**Table 2:** Correct rate of KAST questions (N = 160)

Questions	Correct rate	
	N	%
KAST(1)	93	58.13
KAST(2)	72	45.00
KAST(3)	145	90.63
KAST(4)	116	72.50
KAST(5)	134	83.75
KAST(6)	111	69.38
KAST(7)	116	72.50
KAST(8)	74	46.25
KAST(9)	79	49.38
KAST(10)	132	82.50
KAST(11)	138	86.25
KAST(12)	138	86.25
KAST(13)	112	70.00
KAST(14)	103	64.38
KAST(15)	117	73.13
KAST(16)	67	41.88
KAST(17)	91	56.88

#### Retest reliability

One hundred forty-three caregivers participated in the retest, and the interval between the two surveys was 2–4 weeks. Pearson correlation coefficient analysis was used to test the reliability of the retest.

**Fig. 1:**
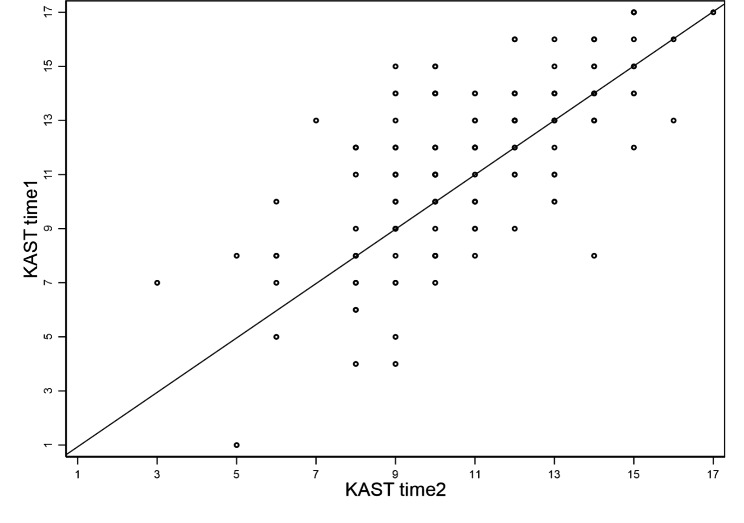
KAST in the first and second applications in 143 caregivers

Figure 1 shows the retest results for these 143 caregivers. The mean score (SD) for the first application was 11.59 (3.08) (range = 1–17), and the mean score (SD) for the second application was 10.92 (2.57) (range = 3–17). The intraclass correlation coefficient was 0.686(p < 0.001).

### Structural validity

The Chinese Mandarin version of KAST was evaluated by factor analysis. as the KMO value is closer to 1, the more common the factors in the variables, and the KMO value for factor analysis should be at least > 0.60. The Chinese Mandarin version of KAST had a KMO value of 0.72 and Bartlett’s sphere test value of 382.240 (p < 0.001), showing the existence of common factors between the overall correlation matrix and was suitable for factor analysis. Principal component analysis was performed on the Chinese Mandarin version of KAST, and five common factors were extracted, which accounted for 52.90% of the total variance. The five common factors were named: management; progression; diagnosis; symptoms; etiology and treatment. Among them, common factor 1 contained questions 3, 4, 5, 13, and 17, common factor 2 contained questions 15 and 16, common factor 3 contained questions 10, 11, and 14, common factor 4 contained 2, 6, and 9, and common factor 5 contained 1, 7, 8, and 12. The results of the structural validity analysis are shown in Table 3.

**Table 3:** The results of the structural validity analysis

Questions	KMO value	Bartlett’s value	Common factor 1	Common factor 2	Common factor 3	Common factor 4	Common factor 5
KAST(1)	0.72	382.240	-0.097	0.136	0.113	0.115	0.664
KAST(2)			-0.033	0.422	0.158	0.580	0.150
KAST(3)			0.601	0.189	0.121	0.059	0.097
KAST(4)			0.643	0.024	0.180	0.018	0.157
KAST(5)			0.478	0.408	0.133	0.028	-0.220
KAST(6)			0.098	0.516	-0.136	-0.616	-0.031
KAST(7)			0.363	0.271	-0.118	0.125	-0.376
KAST(8)			0.090	-0.100	-0.418	0.002	0.590
KAST(9)			0.133	0.038	-0.053	0.775	-0.001
KAST(10)			0.341	0.197	0.560	-0.048	0.099
KAST(11)			0.377	0.123	0.557	-0.137	0.467
KAST(12)			0.299	0.231	0.021	0.170	0.482
KAST(13)			0.424	0.257	0.388	0.319	-0.122
KAST(14)			0.007	0.109	-0.802	-0.123	0.071
KAST(15)			0.025	0.777	0.007	-0.034	0.076
KAST(16)			0.228	0.578	0.054	0.231	0.092
KAST(17)			0.723	-0.018	0.007	0.035	-0.011

### Correlation analysis of KAST score with Sociodemographic and clinical factors

Relationships between KAST Score and caregivers’ sociodemographic and clinical factors in patients with schizophrenia were analyzed (see Table 4). There was a significant correlation between KAST Score and caregivers’ educational attainment (r = 0.179, p = 0.024) and occupational status (r = 0.160, p = 0.044).

**Table 4:** Correlations of KAST Score with Sociodemographic and Clinical factors

Variables	KAST score
CG Age	-0.121
CG Gender	0.105
CG Educational attainment	0.179*
CG Marital status	0.151
Relationship with the patient	-0.118
Occupational status	0.160*
Household financial issues	-0.076
Duration of illness	-0.065
CG: Caregivers Spearman’s rho test was used for all variables *** p < 0.001, ** p < 0.01, * p < 0.05	

### Multivariate linear regression analysis

The mean score of KAST for the 160 caregivers was 11.49 (SD = 3.13). To assess variables as predictors of caregiver KAST score, we built a multivariate linear regression model. Results of multivariate linear regression analyses showed that caregivers’ gender, educational attainment, marital status, relationship with the patient, and occupational status remained statistically significant correlates of KAST score (see Table 5). The overall regression model explained 20.6% of the variation in the KAST score.

**Table 5:** Factors associated with KAST score

KAST score	β	t	p
**CG Age** (ref: 44 or below)	0	.	.
45–54	0.138	0.13	0.899
55–64	1.346	1.13	0.259
65 or above	0.866	0.67	0.504
**CG Gender** (ref: male)	0	.	.
Female	1.004*	2.01	0.047
**CG Educational attainment** (ref: middle school or below)	0	.	.
High school or Junior college	0.832	1.21	0.229
College or above	1.535*	2.54	0.012
**CG Marital status**(ref: Unmarried)	0	.	.
Married	2.267*	2.09	0.038
Other(Divorced/Widowed)	3.568**	2.86	0.005
**Relationship with the patien**t: (ref: Spouse)	0	.	.
Parent	-1.132	-1.62	0.107
Children	− 0.409	-0.40	0.69
Others(e.g., Siblings)	-2.081**	-2.70	0.008
**Occupational status**: (ref: Retired)	0	.	.
Unemployed	2.234*	2.13	0.035
Employed	1.736	1.97	0.051
**Household financial issues**: (ref: Not at all)	0	.	.
Lighter	0.852	1.13	0.261
Fair	0.165	0.25	0.801
More serious	− 0.918	-0.93	0.354
**Duration of illness**: (ref: 14 or below)	0	.	.
15–24	0.839	1.11	0.268
25–34	0.68	0.84	0.4
35 or above	0.318	0.39	0.698
Constant	6.718	3.79	0

*** p < 0.001, ** p < 0.01, * p < 0.05

CG: Caregivers

## Discussion

To the best of our knowledge, this study provided the first cross-cultural adaptation and validation data for the Chinese Mandarin version of KAST, which could be used as a primary assessment of schizophrenia knowledge. The English and Portuguese versions of KAST had been previously validated. Our results showed that the reliability, reproducibility, and validity of the Chinese Mandarin version of KAST were verified based on internal consistency, item analysis, retest reliability, and structural validity. This study also analyzed sociodemographic and clinical factors associated with caregiver KAST scores. The results found gender, educational attainment, marital status, relationship, and occupational status, were each associated with KAST scores.

Our study found that the KAST has sound psychometric properties. We deleted question 17 because of different social backgrounds, patients and their families can obtain relevant information and support for schizophrenia from some formal and more official organizations such as the Chinese Medical Association and the CDC. The Chinese-version KAST had acceptable internal consistency (KR-20 reliability coefficient was 0.702) and reproducibility (intraclass correlation coefficient for retesting was 0.686) through standard translation and screening. Consistent with Compton et al. [10]and the Portuguese version of KAST [11], the original English version had a KR-20 reliability coefficient of 0.82. The Portuguese version had an intraclass correlation coefficient of 0.592 for retesting, both of which verified the validity and reliability of KAST.

A 2014 study in China used a Chinese version of KAST to assess the relationship between knowledge of the illness, medication adherence, and insight in patients with schizophrenia [18]. The translation process of that study was similar to our study, and according to the authors, the Chinese version of KAST removed 4 questions from the original version because they did not match the local reality, and the final Chinese version of KAST had 14 items. Similar to this study, question 17 of the original version was excluded. Similar to the original KAST scale, which contained six domains (cause, symptom, diagnosis, progression, treatment and self-help), the Chinese-Mandarin version of the KAST scale was analyzed for construct validity and five common factors were extracted and named: management (items 3, 4, 5, 13, 17); progression (items 15, 16); diagnosis (items 10, 11, 14); symptom (items 2, 6, 9); etiology and treatment (items 1, 7, 8, 12). The difference was that we grouped item 3 ‘Best person to diagnose schizophrenia’, item 4 ‘Most common long-term consequences of schizophrenia’ and item 5 ‘Medication for hallucinations’ into the common factor ‘Management’. In our study, items 2, 8, 9, and 16 were less than 50% correct regarding the etiology and symptoms of schizophrenia and atypical drugs used for treatment, indicating that they still have misconceptions about the etiology and symptoms of schizophrenia and lack of knowledge about the atypical drug " Quetiapine” used for treatment. In addition, perceiving the problem of schizophrenia etiology as non-biological may reflect the general health beliefs of the Chinese [19]; caregivers are more likely to attribute the etiology of schizophrenia to psychological or supernatural causes [20].

Multivariate regression results indicated that female caregivers had better knowledge of the illness (higher KAST scores) than males. This is consistent with previous findings, which suggested female caregivers gained higher levels of knowledge than male caregivers after participating in psychoeducation programs, but it may be related to the relatively higher educational attainment of women participating in psychoeducation [21]. Also, in our study, individuals with at least a university education had a higher level of knowledge about the disease compared to caregivers with a middle school education or below. This is consistent with the results of the English [10]and Portuguese [11] versions of the test while reinforcing, to some extent, the validity of the instrument’s construct, theoretically, those college-educated individuals have higher levels of knowledge. Caregivers who are married or divorced or widowed tend to be older and may have a better understanding of the disease due to the longer time spent caring for the patient. For unemployed caregivers, they may also have a better understanding of the disease because they have lost their jobs and have more effort to care for the patient. Conversely, caregivers who are in other relationships such as siblings may also have a poorer understanding of the disease because they may be less close to the patient in comparison to parents or children. Tools such as KAST to assess knowledge of schizophrenia may be important in developing rehabilitation intervention programs for patients with schizophrenia, increasing awareness of schizophrenia, and reducing patient stigma. For caregivers of patients with schizophrenia, promoting caregivers’ knowledge of schizophrenia can help them become better familiar with the etiology, symptoms, treatment, and rehabilitation of the disorder, thereby reducing prejudice and increasing their understanding of patients with schizophrenia, among other things.

This study is subject to several limitations. First, we selected participants from community health centers by convenience sampling, which may not be representative of the general schizophrenia patients and their caregivers. Second, the KAST may be more appropriate for measuring knowledge in the general population, and caregivers of people with schizophrenia tend to have better knowledge of schizophrenia, further validity analysis of the Chinese version of KAST in the general population is warranted. Another limitation is that although KAST is a good psychometric questionnaire, not all reliability and validity testing methods can be used. Compton et al [10] also pointed out that the lack of a gold standard for measuring knowledge about schizophrenia makes it difficult to assess the standard validity of KAST.

## Conclusion

Our study demonstrates that the Chinese version of KAST with standardized translation and screening is valid and reproducible when testing caregivers’ knowledge of schizophrenia and can be used as a knowledge assessment tool for rehabilitation interventions and psychoeducational interventions. Given that our study found that caregiver gender, educational attainment, marital status, relationship, and occupational status were correlated with the level of illness knowledge, these sociodemographic characteristics should be considered when using the tool. Further use of this tool to assess knowledge of schizophrenia may be considered in related studies in the future.

## Data Availability

The datasets generated and/or analyzed during the current study are not publicly available due to privacy regulations but are available from the corresponding author upon reasonable request.

## Abbreviations

KAST: Knowledge About Schizophrenia Test
ICC: Intraclass correlation coefficient
CG: Caregivers
